# Asymmetric Airfoil Morphing via Deep Reinforcement Learning

**DOI:** 10.3390/biomimetics7040188

**Published:** 2022-11-03

**Authors:** Kelin Lu, Qien Fu, Rui Cao, Jicheng Peng, Qianshuai Wang

**Affiliations:** 1 School of Automation, Southeast University, Nanjing 210096, China; 2 College of Information Engineering (Artificial Intelligence), Yangzhou University, Yangzhou 225009, China; 3 College of Automation Engineering, Nanjing University of Aeronautics and Astronautics, Nanjing 211106, China; 4 School of Electrical Engineering, North China University of Science and Technology, Tangshan 063210, China

**Keywords:** airfoil morphing, shape memory alloys, hysteresis, deep reinforcement learning

## Abstract

Morphing aircraft are capable of modifying their geometry configurations according to different flight conditions to improve their performance, such as by increasing the lift-to-drag ratio or reducing their fuel consumption. In this article, we focus on the airfoil morphing of wings and propose a novel morphing control method for an asymmetric deformable airfoil based on deep reinforcement learning approaches. Firstly, we develop an asymmetric airfoil shaped using piece-wise Bézier curves and modeled by shape memory alloys. Resistive heating is adopted to actuate the shape memory alloys and realize the airfoil morphing. With regard to the hysteresis characteristics exhibited in the phase transformation of shape memory alloys, we construct a second-order Markov decision process for the morphing procedure to formulate a reinforcement learning environment with hysteresis properties explicitly considered. Subsequently, we learn the morphing policy based on deep reinforcement learning techniques where the accurate information of the system model is unavailable. Lastly, we conduct simulations to demonstrate the benefits brought by our learning implementations and validate the morphing performance of the proposed method. The simulation results show that the proposed method provides an average 29.8% performance improvement over traditional methods.

## 1. Introduction

While unmanned aerial vehicles (UAVs) have played a crucial role in various civil and military missions, studies demonstrate that birds usually possess higher flight maneuverability and agility than comparatively-sized aircraft in complex and varying environments [1,2]. One of the critical advantages of birds is that they morph their wings and tails intricately to perform efficient behaviors including perching, hovering and maintaining stability under different flight conditions [3]. Such aerodynamic adaptability has aroused flourishing interest in the design and control of avian-inspired morphing UAVs [4,5,6]. In this work, we focus on aircraft capable of morphing their wings by modifying the geometric configuration of the airfoil shape, which refers to more specifically camber morphing [7]. Bird wings are usually cambered to generate sufficient lift force at a low angle of attack. The camber does not remain constant through their flight, and many observations show that birds actively control the camber of their proximal wing via remiges and modify the distal wing airfoil shape via their primary feathers [8,9]. These investigations provide insight into the study of airfoil-morphing aircraft. The benefits brought by camber morphing for aircraft include increasing lift, reducing drag and airframe noise mitigation [10]. Applications of camber morphing mechanisms include a lead-edge morphing combined smart droop nose design, which achieves high-lift performance with significantly reduced complexity and mass [11], and a flexible morphing trailing edge design with deformable ribs, which is used to enhance the Fowler flaps and act as a substitution for ailerons for civil transport aircraft [12].

The ideal airfoil is usually generated using shape optimization techniques including gradient-based methods and gradient-free methods [13], which optimize some aerodynamic performance parameters of aircraft, such as the drag coefficient, the lift coefficient and the lift-to-drag ratio [14,15,16]. Recently, deep reinforcement learning (DRL) approaches such as proximal policy optimization (PPO) [17] have been exploited to learn the airfoil shape directly according to performance metrics computed by computational fluid dynamics (CFD) solvers [18]. In [19], a 3D-printed morphing airfoil model is developed, and the optimal configuration is generated via Q-learning to match the desired pitching moment. In [20], the transfer learning technique is combined with DRL for shape optimization, which formulates a multi-fidelity framework and reduces the computational cost.

Although an optimized airfoil shape can be calculated given a certain flight condition, it is challenging to morph into optimal shapes instantaneously during the flight procedure due to the uncertainty and inconsistency of the environments and aircraft dynamics [2,21]. This galvanizes the utilization of data-driven methods including deep learning and reinforcement learning for morphing control. In [22], the morphing air vehicle was modeled as a smart block in the shape of a rectangular parallelopiped, and the control policy was learned by actor-critic methods. This framework was extended to an ellipsoid-shaped aircraft with Q-learning methods to produce a more efficient policy [23]. The thickness and camber of the airfoil shape were adjusted via Q-learning in [24], where the rewards were related to aerodynamic parameters. The constant-strength source doublet panel method was utilized in [25] to calculate the aerodynamic forces on the morphing air vehicle. In [26], airfoil morphing by vertically moved control points was designed, where the unknown drag and lift coefficients were estimated using neural networks.

Concerning the realization of morphing mechanisms, biologically inspired mechanical joints are often adopted to control sweep-, dihedral- and twist-morphing wings [27,28,29]. However, the development and actuation for camber-morphing aircraft are more challenging, as they are required to permit smooth transitions in the airfoil shape [2]. Conventional actuators for camber morphing include servo motors and hydraulic actuators [30,31], where the wing is constructed by articulated rigid-linked components [32] or a deformable skin with internal compliant mechanisms [33]. Recent advances in material technologies have led to a proliferation of applications of smart materials, especially shape memory alloys (SMA), for morphing aircraft [34,35]. Shape memory alloys are metallic alloys capable of transforming their crystalline structures between two phases to deform and recover their shapes through heating and cooling, which is suitable for morphing actuators because of their properties, including high-actuation energy densities and large recoverable strains [35]. A morphing wing under subsonic cruise flight conditions was developed in [36] using a flexible skin and a group of SMA actuators, which reduced the fuel consumption. In [37], the authors designed an autonomous morphing helicopter rotor blade where the SMAs equipped on the blade modify the section camber according to ambient temperature. In [38], a super critic airfoil actuated by SMAs was developed, and the transonic aerodynamics are investigated. Nevertheless, to actively control the strain of SMA wires is not straightforward due to the temperature hysteresis exhibited during the phase transformation [39]. This gives rise to increasing investigations of learning-based control for SMAs. Reinforcement learning methods including Q-learning and Sarsa have been applied to adjust the strain of SMAs via resistive heating, where the hysteretic dynamics are modeled as hyperbolic tangent curves [40,41]. However, there is no explicit consideration for the hysteresis properties in the policy-learning procedure of these studies.

In this work, the morphing control for a deformable asymmetric airfoil based on deep reinforcement learning techniques is investigated. The contributions of this work are threefold, as follows. Firstly, the shape of the asymmetric airfoil is designed based on Bézier curves, and the morphing mechanism of such airfoils is modeled via SMA wires. Subsequently, a dynamic system between input voltages and airfoil shapes is developed, which characterizes the hysteresis behaviors in the manner of Markov decision processes. Finally, the morphing policy is constructed based on deep reinforcement learning approaches without accurate knowledge of system models, which adjusts the airfoils during flight procedures to track the optimal shapes in different flight conditions.

The rest of this paper is organized as follows. In Section 2, the morphing method is developed in three steps. In Section 2.1, the asymmetric airfoil is shaped using piece-wise Bézier curves. In Section 2.2, the morphing airfoil is modeled by SMAs, and a dynamic model is derived. In Section 2.3, a deep reinforcement learning-based morphing policy is developed. In Section 3, simulations are conducted to validate the proposed methods. In Section 4, this work is summarized.

## 2. Materials and Methods

### 2.1. Asymmetric Airfoil Shape Modeling

Well-known methods of curve synthesis for airfoil design and optimization include splines (e.g., B-spline and Bézier curves) [42,43], free-form deformation (FFD) [44], and class-shape transformations (CST) [45]. In this work, Bézier curves are selected for their straightforward design procedure and simple calculations. The shape of the asymmetric airfoil is parameterized via *N* control points, which are connected by Bézier curves [18,20]. To generate an untangled shape, the control points are distributed in an annulus with a predefined inner radius $R_{1}$ and outer radius $R_{2}$. Moreover, the annulus is partitioned into *N* sectors equally, in each of which a control point is placed. These points are sorted with respect to the azimuth and denoted as $p_{i}\in R^{2}$ for i=1,⋯,N in Cartesian coordinates. Each pair of adjacent points is augmented by two other points and then connected via a cubic Bézier curve. For each control point, $p_{i}$, an auxiliary angle, is calculated to determine the tangent to the curve at this point, which is given by

(1) $$\theta_{i}^{*}=\alpha_{i}\theta_{i-1,i}+\left(1-\alpha_{i}\right)\theta_{i,i+1}$$

where $\theta_{i,i+1}$ is the angle between point $p_{i}$ and $p_{i+1}$, and $\alpha_{i}\in \left[0,1\right]$ is an averaging parameter to modify the local smoothness of the curve. Then, two augmented points for the curve between $p_{i}$ and $p_{i+1}$ are calculated by

(2) $$\begin{array}{cc} p_{i}^{*} & =p_{i}+\eta_{i}∥p_{i+1}-p_{i}∥\cdot e_{i}\\ p_{i}^{**} & =p_{i+1}-\eta_{i}∥p_{i+1}-p_{i}∥\cdot e_{i+1} \end{array}$$

where $e_{i}={\left[\cos\left(\theta_{i}^{*}\right),\sin\left(\theta_{i}^{*}\right)\right]}^{⊤}$, and the scale parameter $\eta_{i}$ controls the local curvature. The curve connecting $p_{i}$ and $p_{i+1}$ is given by

(3) $$b\left(t\right)={\left(1-t\right)}^{3}p_{i}+3{\left(1-t\right)}^{2}tp_{i}^{*}+3\left(1-t\right)t^{2}p_{i}^{**}+t^{3}p_{i+1},0\leq t\leq 1$$

**Figure 1:**
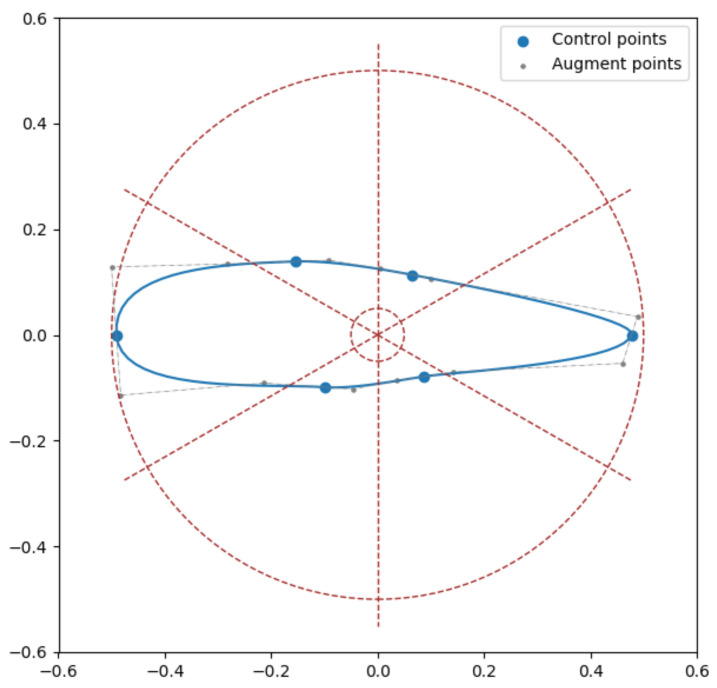
An illustration of a valid airfoil shape with six control points. The dashed circles denote the minimum and maximum radius for each point. The dashed rays split the annulus into *N* equal sections.

An example of a valid shape is illustrated in Figure 1. The morphing wings in this work take NACA-2424 [46,47] as the baseline airfoil.

### 2.2. Dynamic System of Airfoil Morphing

Denote the polar coordinate of each point $p_{i}$ as ${\left\{r_{i},\theta_{i}\right\}}_{i=1}^{N}$. Then the shape of the airfoil is fully determined by the radius $r_{i}$, the angle $\theta_{i}$ and the auxiliary angle parameters $\alpha_{i}$, $\eta_{i}$. In the flight procedure of the aircraft, we aim to morph the airfoil to maximize desired aerodynamic performances, such as the lift-to-drag ratio $C_{l}/C_{d}$, according to various conditions, including flight position, velocity or attack angle. With the combination of CFD solvers and optimization methods, the preferred airfoil shape at a given flight condition can be determined previously. Such optimized shapes serve as the reference or target airfoil shapes for the morphing task. Then, the problem of how to control the airfoil to achieve optimized shapes during the flight procedure where the flight condition varies is investigated.

Since there has been a variety of investigations on the position control of DC motors [48], we assume that the polar angle of each control point tracks the reference trajectory well via motors. Additionally, we assume that the auxiliary angle parameters can be adjusted rapidly and accurately. Therefore, in this work, we focus on the optimal morphing in the aspect of modifying the radii of control points.

Smart materials, especially shape memory alloys (SMA), are adopted to realize the airfoil morphing, where the radii of control points are modified via adjusting the length of SMA wires. Firstly, the dynamic model is constructed between the wire temperature and radii. An SMA wire changes its length through the crystal phase transformation between martensite and austenite according to the temperature. The transitions to martensite and austenite have different start and end temperatures, which leads to the hysteresis properties of the strain with respect to the temperature. Instead of common methods such as Preisach model and Krasnosel’skii–Pokrovskii model [49], the SMA hysteresis is characterized using hyperbolic tangent functions for their efficiency in computation and accuracy in curve fitting [41]. The strain is replaced by a radius factor $\gamma_{i}=\left(r_{i}-R_{1}\right)/\left(R_{2}-R_{1}\right)$ equivalently such that $\gamma_{i}\in \left[0,1\right]$. For heating and cooling starting with temperatures outside the transformation region, namely that the initial temperature is not between the end temperatures of the phase transformations, the hysteresis properties are modeled by the major hysteresis loops as

(4) $$f_{l}^{major}\left(T\right)=\frac{h_{0}}{2}\tanh\left(\left(T-c_{tl}\right)c_{b}\right)+w\left(T-\frac{c_{tl}+c_{tr}}{2}\right)+\frac{h_{0}}{2}+c_{s}$$

(5) $$f_{r}^{major}\left(T\right)=\frac{h_{0}}{2}\tanh\left(\left(T-c_{tr}\right)c_{b}\right)+w\left(T-\frac{c_{tl}+c_{tr}}{2}\right)+\frac{h_{0}}{2}+c_{s}$$

where *T* denotes the temperature, and $h_{0}$, $c_{tl}$, $c_{tr}$, $c_{b}$, *w*, $c_{s}$ parameterize the shape of the curves. Values of such parameters are chosen to fit the experimental data of SMA [40,41]. The radius factor γ varies according to the $f_{l}^{major}$ curve when the temperature decreases and $f_{r}^{major}$ when the temperature increases. Furthermore, switching the temperature direction during the transformation procedure causes a reverse transformation starting from the current temperature and strain, which is not on the major loop of the reverse transformation. Such transforms are modeled using minor hysteresis loops, which are modeled by hyperbolic tangent curves with similar shapes as the major loops. The function of a rising minor loop is given as

(6) $$f_{r}^{minor}\left(T,h\right)=\frac{h}{2}\tanh\left(\left(T-c_{tr}\right)c_{b}\right)+w\left(T-\frac{c_{tl}+c_{tr}}{2}\right)+h_{0}-\frac{h}{2}+c_{s}$$

where *h* is selected to ensure the intersection of the consecutive curves at the current point and is given by

(7) $$h=g_{r}\left(h_{\mathrm{prev}},T\right)=\frac{h_{\mathrm{prev}}\left(\tanh\left(\left(T-c_{tl}\right)c_{b}\right)+1\right)-2h_{0}}{\tanh\left(\left(T-c_{tr}\right)c_{b}\right)-1}$$

and $h_{\mathrm{prev}}$ is the height parameter of the previous curve. Functions for the lowering curves are analogous as

(8) $$f_{l}^{minor}\left(T,h\right)=\frac{h}{2}\tanh\left(\left(T-c_{tl}\right)c_{b}\right)+w\left(T-\frac{c_{tl}+c_{tr}}{2}\right)+\frac{h}{2}+c_{s}$$

and

(9) $$h=g_{l}\left(h_{\mathrm{prev}},T\right)=\frac{h_{\mathrm{prev}}\left(\tanh\left(\left(T-c_{tr}\right)c_{b}\right)-1\right)+2h_{0}}{\tanh\left(\left(T-c_{tl}\right)c_{b}\right)+1}$$

**Figure 2:**
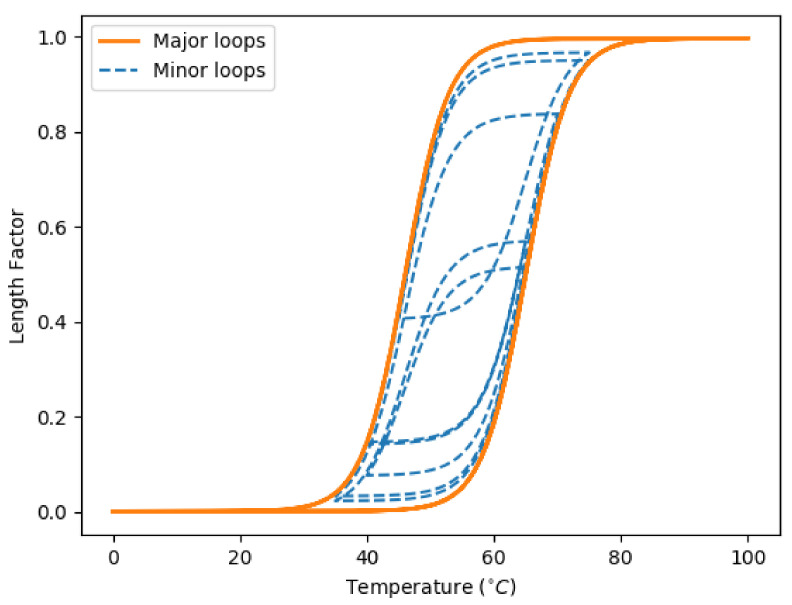
An illustration on the hysteresis loops of SMA wires.

An illustration of the transformation procedure is given in Figure 2.

**Figure 3:**
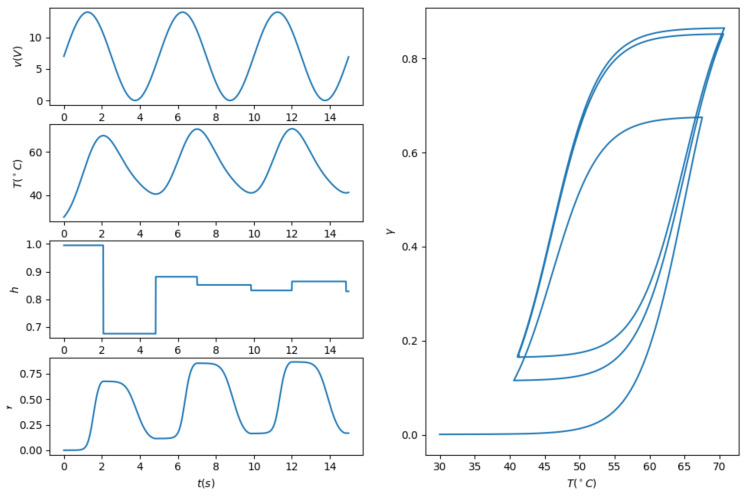
An illustration on the dynamics of SMA wires with sinusoidal voltage input.

After constructing the temperature-strain model, resist heating is used to actuate the SMA wires [50]. Given the applied voltage $v_{i}$, the temperature *T* follows the heat transfer model [39]

(10) $$m_{w}c_{w}\dot{T_{i}}=\frac{v_{i}^{2}}{R_{w}}-h_{w}A_{w}\left(T_{i}-T_{f}\right)\;i=1,\cdots ,N$$

where $m_{w}$ is the mass per unit length of the SMA wire, $c_{w}$ is the specific heat, $R_{w}$ is the electrical resistance per unit length, $h_{w}$ is the heat exchange coefficient, $A_{w}$ is the wire circumferential area, and $T_{f}$ is the airflow temperature. Combining the temperature-strain relationship and (10), it is shown that the dynamic system between the radius and the input voltage are highly nonlinear due to the hysteresis characteristics. An illustration on the dynamics of SMA wires driven by a sinusoidal voltage input is given in Figure 3.

In this work, we tackle the morphing problem for the airfoil constructed by the SMA wires whose dynamics are given in (4)–(10). Note that the temperature-strain and voltage-temperature relationships modeled above are not directly accessible to our controller, but serve as the environment from which paths of the states can be sampled. We resort to deep reinforcement learning methods to design the morphing policy.

### 2.3. Reinforcement Learning based Morphing Control

Reinforcement learning (RL) methods are capable of learning a control policy from interactions between the given agent and environment, with no requirement on the knowledge of system models [51]. The learning procedures are based on Markov decision processes (MDPs), which are given by 4-tuples $\left\{S,A,R,P\right\}$, where *S* and *A* are the state space and action space containing all the states and actions, respectively, *R* is the reward function giving $r_{k}=R\left(s_{k},a_{k},s_{k+1}\right)$ as rewards, and *P* is the transition function giving $P\left(s_{k+1}|s_{k},a_{k}\right)$ as state transition probabilities. In this section, the airfoil morphing problem is solved in the RL framework, where we aim to find the optimal policy maximizing the expected total rewards.

Before choosing the states and actions for the morphing problem, the morphing system is investigated further to construct an MDP from it. Firstly, the voltage-temperature dynamics (10) of SMA wires is discretized via Euler methods as

(11) $$T_{i,k}=T_{i,k-1}+\Delta t\cdot \sigma \left(T_{i,k-1},v_{i,k-1}\right)$$

where Δt>0 is the discretizing time step, and

(12) $$\sigma \left(T,v\right)=\frac{v^{2}}{m_{w}c_{w}R_{w}}-\frac{h_{w}A_{w}}{m_{w}c_{w}}\left(T-T_{f}\right)$$

With regard to the temperature-strain dynamics, since the minor loops converge with the major loops outside the SMA’s transformation region, we assume that the major loops determine the initial states of the wires, and minor loops dominate the dynamics during the morphing procedure. We denote the function (8) as $f_{l}\left(T,h\right)$ and (6) as $f_{r}\left(T,h\right)$ for simplicity, and introduce the signum function

(13) $$\mathrm{sgn}\left(x\right)=\left\{\begin{array}{c} 1,x\geq 0\\ 0,x<0 \end{array}\right.$$

Then, $\mathrm{sgn}(T_{k}-T_{k-1})$ is used to discriminate the status of raising or lowering the temperature at time *k*. According to (11) and the fact that Δt>0, we describe the temperature direction at time *k* by

(14) $$\chi_{i,k}≜\mathrm{sgn}\left(T_{k}-T_{k-1}\right)=\mathrm{sgn}\left(\sigma \left(T_{i,k-1},v_{i,k-1}\right)\right)$$

Note that the strain is dependent on the height parameter of the current loop. Recall from (7) and (9) that the value of the height parameter changes when the direction of temperature switches. Then, the time-varying parameter is determined by

(15) $$\begin{array}{cc} h_{i,k}= & \left(1-\chi_{i,k-1:k}\right)\cdot \left(\chi_{i,k}g_{r}\left(T_{i,k},h_{i,k-1}\right)+\left(1-\chi_{i,k}\right)g_{l}\left(T_{i,k},h_{i,k-1}\right)\right)\\ +\chi_{i,k-1:k}h_{i,k-1} \end{array}$$

where

(16) $$\chi_{i,k-1:k}≜\mathrm{sgn}\left(\sigma \left(T_{i,k-2},v_{i,k-2}\right)\sigma \left(T_{i,k-1},v_{i,k-1}\right)\right)$$

detects the reversal of temperature direction. Subsequently, the radius factor given the temperature and height parameter is calculated by choosing the rising or lowering loop according to the current temperature direction as

(17) $$\gamma_{i,k}=\chi_{i,k}f_{r}\left(T_{i,k},h_{i,k}\right)+\left(1-\chi_{i,k}\right)f_{l}\left(T_{i,k},h_{i,k}\right)$$

Summarizing the relationships (11), (15) and (17), it seems appropriate to select *T* and *h* as states and *v* as actions to construct a RL environment for airfoil morphing. The radius factor can be treated as an observation since it is dependent on only the current voltage, temperature and height parameter. Since the length and temperature of SMA wires can be measured directly via sensors equipped on the airfoils, these values are assumed to be available. However, the height parameter is not a realistic physical characteristic but just a coefficient fitting the hyperbolic tangent curves to the actual SMA properties, which makes the measurement on *h* not available. Actually, we aim to adjust the position of control points to achieve a reference airfoil shape, such that we want to learn a policy on modifying the lengths (i.e., the radius factors). We observe from the loop functions (6) and (8) that, given the temperature *T*, the function of γ with respect to *h* is bijective. Therefore, denoting the relationship (17) as $\gamma_{i,k}=f\left(h_{i,k},T_{i,k},T_{i,k-1},v_{i,k-1}\right)$, we can obtain an inverse function as

(18) $$h_{i,k}=f^{-1}\left(\gamma_{i,k},T_{i,k},T_{i,k-1},v_{i,k-1}\right)$$

Combining (15) and (18), the dynamics of radius factors are described as

(19) $$\begin{array}{cc} \gamma_{i,k}= & f((1-\chi_{i,k-1:k})\cdot [\chi_{i,k}g_{r}\left(T_{i,k},f^{-1}(\gamma_{i,k-1},T_{i,k-1},T_{i,k-2},v_{i,k-2})\right)\\ & +\left(1-\chi_{i,k}\right)g_{l}\left(T_{i,k},f^{-1}(\gamma_{i,k-1},T_{i,k-1},T_{i,k-2},v_{i,k-2})\right)]\\ & +\chi_{i,k-1:k}f^{-1}(\gamma_{i,k-1},T_{i,k-1},T_{i,k-2},v_{i,k-2}),T_{i,k},T_{i,k-1},v_{i,k-1}) \end{array}$$

Note that $\chi_{i,k-1:k}$ is dependent on the temperature and voltage at time k−2. This makes the transition function of γ a second-order difference equation.

**Proposition** **1.** *Given a second-order MDP with states $s^{\prime }\in R^{n_{s^{\prime }}}$ and actions $a^{\prime }\in R^{n_{a^{\prime }}}$ satisfying*

(20) $$p\left(s_{k}^{\prime }|s_{k-1}^{\prime },a_{k-1}^{\prime },s_{k-2}^{\prime },a_{k-2}^{\prime },\ldots ,s_{0},a_{0}\right)=p\left(s_{k}^{\prime }|s_{k-1}^{\prime },a_{k-1}^{\prime },s_{k-2}^{\prime },a_{k-2}^{\prime }\right)\;k\geq 2$$

*select $s_{k}={\left[s_{k}^{\prime ⊤},s_{k-1}^{\prime ⊤}\right]}^{⊤}$ and $a_{k}={\left[a_{k}^{\prime ⊤},a_{k-1}^{\prime ⊤}\right]}^{⊤}$. Then, an MDP can be constructed by states s and actions a (with an additionally defined state transition function and reward function). Moreover, if the second-order MDP with $s^{\prime }$ and $a^{\prime }$ has a deterministic state transition function*

(21) $$s_{k}^{\prime }=f_{s}\left(s_{k-1}^{\prime },a_{k-1}^{\prime },s_{k-2}^{\prime },a_{k-2}^{\prime }\right)$$

*then the MDP constructed by s and a satisfies the transition function*

(22) $$s_{k}={\left[f_{s}^{⊤}\left(\left[I_{n_{s^{\prime }}},0\right]s_{k-1},\left[I_{n_{a^{\prime }}},0\right]a_{k-1},\left[0,I_{n_{s^{\prime }}}\right]s_{k-1},\left[0,I_{n_{a^{\prime }}}\right]a_{k-1}\right),{\left(\left[0,I_{n_{s^{\prime }}}\right]s_{k-1}\right)}^{⊤}\right]}^{⊤}$$

This proposition can be derived directly via the properties of MDPs. According to Equations (11), (19) and Proposition 1, it is reasonable to choose the radius factors $\left\{\gamma_{i,k-1},\gamma_{i,k}\right\}$ and temperatures $\left\{T_{i,k-1},T_{i,k}\right\}$ as states with input voltages $\left\{v_{i,k-1},v_{i,k}\right\}$ as actions. We restrict the states by $\gamma \in \left[0,1\right]$ and the actions by $v\in \left[0,V_{\max}\right]$.

The reference radius factors of the optimized airfoil shape under certain condition $c_{k}$ are denoted as $\gamma_{i,k}^{\mathrm{ref}}\left(c_{k}\right)$. When determining the reward, we expect the airfoil to morph to reference shapes accurately and rapidly. Therefore, a sparse reward function comprised of two components at time *k* is designed by

(23) $$r_{k}^{\prime }=R\left(s_{k},a_{k},c_{k}\right)=\sum_{i=1}^{N}\left(r_{k,i}^{\mathrm{pos}}+r_{k,i}^{\mathrm{vol}}\right)$$

where the position reward conveying the requirement of morphing accuracy is given by

(24) $$r_{k,i}^{\mathrm{pos}}=\left\{\begin{array}{c} r_{p},\;\left|\gamma_{i,k}-\gamma_{i,k}^{\mathrm{ref}}\left(c_{k}\right)\right|\leq e_{\mathrm{thr}}\\ 0,\;|\gamma_{i,k}-\gamma_{i,k}^{\mathrm{ref}}\left(c_{k}\right)|>e_{\mathrm{thr}} \end{array}\right.$$

and the voltage reward aiming to increase the morphing speed is given by

(25) $$r_{k,i}^{\mathrm{vol}}=\left\{\begin{array}{c} r_{v},\;\left|\gamma_{i,k}-\gamma_{i,k}^{\mathrm{ref}}\left(c_{k}\right)\right|>e_{\mathrm{thr}}\;\mathrm{and}\;\mathrm{sgn}\left(v_{i,k}-v_{i,k-1}\right)=\mathrm{sgn}\left(\gamma_{i,k}^{\mathrm{ref}}\left(c_{k}\right)-\gamma_{i,k}\right)\\ 0,\;\mathrm{otherwise} \end{array}\right.$$

where $r_{p}>r_{v}>0$ and $e_{\mathrm{thr}}>0$ are tunable hyperparameters. When the position error is small, the voltage reward is eliminated to mitigate the oscillation. The choice of adjacent voltages as actions permits the calculation of the voltage reward in the MDP framework. Furthermore, the total return to be maximized is given by

(26) $$R\left(\tau \right)=\sum_{k=0}^{K}r_{k}^{\prime }$$

where $\tau =\left(s_{0},a_{0},c_{0},s_{1},a_{1},c_{1}\ldots \right)$ denotes the sequence of states, actions and conditions.

After establishing the MDP, we proceed to tackle the morphing task based on deep reinforcement learning techniques. Our learning method is designed based on the soft actor-critic (SAC) algorithm [52], which is an off-policy reinforcement learning method compatible with continuous state and action spaces. In the actor-critic framework, the agent learns to interact with the environment and obtain maximum rewards via training two types of neural networks iteratively. The first one is named a critic network and accepts current states and actions as input to approximate the action-value function, which serves as an evaluation of the current policy. The second one is denoted as an actor network and generates actions according to the system states and optional external inputs. After the training is converged, the policy is determined by the actor network and conducted for online executions, which in this work is the morphing task. In SAC, a stochastic policy is learned with additional entropy regularization in the rewards, which improves the ability of exploration and achieves faster convergence for a variety of control problems. According to the MDP of the morphing procedure, we choose the state $s_{k}$ to be $s_{k}=\left[s_{1,k-1}^{\prime },s_{1,k}^{\prime },\ldots ,s_{N,k-1}^{\prime },s_{N,k}^{\prime }\right]$ and the action as $a_{k}=\left[a_{1,k-1}^{\prime },a_{1,k}^{\prime },\ldots ,a_{N,k-1}^{\prime },a_{N,k}^{\prime }\right]$, where $s_{i,k}^{\prime }=\left[\gamma_{i,k},T_{i,k}\right]$ and $a_{i,k}^{\prime }=v_{i,k}$. Note that since the reference airfoil shapes guide the morphing, the flight condition should be incorporated for the generation and evaluation of the actions. We denote the distribution of the stochastic policy as π(a|s,c).

With regard to the construction of the action-value function, instead of directly applying (23), we augment the reward with policy entropy as

(27) $$r_{k}=R\left(s_{k},c_{k},a_{k}\right)=\sum_{i=1}^{N}\left(r_{k,i}^{\prime }+\beta H_{\pi }\left(s_{i,k},c_{k}\right)\right)$$

where

(28) $$H_{\pi }\left(s_{i,k},c_{k}\right)=E_{a_{i,k}∼\pi \left(\cdot |s_{i,k},c_{k}\right)}\left[-\log\pi (a_{i,k}|s_{i,k},c_{k})\right]$$

is the entropy representing the randomness of the policy, and β>0 is the trade-off coefficient. According to (26), we define a finite-horizon undiscounted return to be maximized. Nevertheless, a discount factor is applied when evaluating the value functions to focus on recent rewards, since the future reference shapes are not accessible at the current time step. Then, the action-value function with the regularized reward function is introduced as

(29) $$Q^{\pi }\left(s,a,c,k\right)=E_{\tau ∼\pi }\left[-\sum_{t=k}^{K}\sum_{i=1}^{N}\xi^{t}r_{k,i}^{\prime }+\sum_{t=k+1}^{K}\sum_{i=1}^{N}\xi^{t}\beta H_{\pi }\left(s_{i,t},c_{t}\right)|s_{k}=s,a_{k}=a,c_{k}=c\right]$$

Afterwards, the Bellman equation for the action-value function is given as

(30) $$\begin{array}{cc} Q^{\pi } & (s_{k},a_{k},c_{k},k)=\\ & r_{k}+E_{s_{k+1}∼P,a_{k+1}∼\pi }\left[\xi \left(Q^{\pi }\left(s_{k+1},a_{k+1},c_{k+1},k+1\right)-\beta \log\pi \left(a_{k+1}|s_{k+1},c_{k+1}\right)\right)\right] \end{array}$$

and following the schedule of SAC [52], we approximate the expectation by

(31) $$Q^{\pi }\left(s_{k},a_{k},c_{k},k\right)\approx r_{k}+\xi \left(Q^{\pi }\left(s_{k+1},{\tilde{a}}_{k+1},c_{k+1},k+1\right)-\beta \log\pi \left({\tilde{a}}_{k+1}|s_{k+1},c_{k+1}\right)\right)$$

where $\left\{s_{k},a_{k},c_{k},s_{k+1},c_{k+1}\right\}$ are sampled from replay buffers, and the next action ${\tilde{a}}_{k+1}$ is sampled from the current policy $\pi (\cdot |s_{k+1},c_{k+1})$.

For the learning of the action-value functions, the double-Q trick is applied to avoid overestimation [53]. Two critic networks for Q functions are implemented as $Q_{\phi_{1}}\left(s,a,c,k\right)$ and $Q_{\phi_{2}}\left(s,a,c,k\right)$, where $\phi_{1}$ and $\phi_{2}$ are parameters. Additionally, for the stabilization of the training procedure, the target networks $Q_{\phi_{1}^{\prime }}$ and $Q_{\phi_{2}^{\prime }}$, which are copies of $Q_{\phi_{1}}$ and $Q_{\phi_{2}}$, are used and updated by polyak averaging after each time we update the main critic networks as

(32) $$\phi^{\prime }\leftarrow \rho \phi^{\prime }+\left(1-\rho \right)\phi$$

where ρ∈(0,1) is the update hyperparameter. Summarizing all these settings, the loss for critic networks is given by the mean squared Bellman error function as

(33) $$L\left(\phi \right)=\sum_{b\in B}\left(Q_{\phi }\left(s_{k},a_{k},c_{k},k\right)-y\left(s_{k+1},a_{k+1},c_{k+1},k+1\right)\right)$$

where *B* is the sampled batch with elements $b=\left\{s_{k},a_{k},c_{k},r_{k},k,s_{k+1},c_{k+1},k+1\right\}$ of the replay buffer, and where

(34) $$y\left(s_{k+1},a_{k+1},c_{k+1},k+1\right)=\xi \left(Q_{\phi^{\prime }}\left(s_{k+1},{\tilde{a}}_{k+1},c_{k+1},k+1\right)-\beta \log\pi \left({\tilde{a}}_{k+1}|s_{k+1},c_{k+1}\right)\right)$$

is calculated using target networks. Stochastic gradient descent is applied to update $\phi_{1}$ and $\phi_{2}$ with respect to the loss functions $L(\phi_{1})$ and $L(\phi_{2})$.

Subsequently, we aim to find the policy that maximizes the expected action-value function with respect to the actions. Denote the parameters of actor network as θ. Since the action at time step *k* is composed of the input voltages at k−1 and *k*, the actor network should be designed as $\pi_{\theta }\left(s_{k},c_{k},a_{k-1}\right)$, where an identity layer is applied to propagate the previous voltages. However, the distribution of new input voltages is still only dependent on $s_{k}$ and $c_{k}$. Therefore, we use $\pi_{\theta }\left(a_{k}|s_{k},c_{k}\right)$ to denote the density of action $a_{k}$. Then, the value function to be optimized is given as

(35) $$V^{\pi_{\theta }}\left(s_{k},c_{k},k\right)=E_{a_{k}∼\pi_{\theta }}\left[Q^{\pi_{\theta }}\left(s_{k},a_{k},c_{k},k\right)-\beta \log\left(\pi_{\theta }\left(a_{k}|s_{k},c_{k}\right)\right)\right]$$

The reparameterization trick is adopted here for the sake of the efficient computation of gradients [54]. We introduce a standard normal distributed variable $\zeta ∼p\left(\zeta \right)=N\left(0,I_{n_{a}}\right)$ and calculate the input voltage according to a deterministic squashing function as

(36) $$v_{k}=\frac{V_{\max}}{2}\left(1+\tanh\left(\mu_{\theta }\left(s_{k},c_{k}\right)+\sigma_{\theta }\left(s_{k},c_{k}\right)\circ \zeta_{k}\right)\right)$$

where $\mu_{\theta }\left(s,c\right)$ and $\sigma_{\theta }\left(s,c\right)$ are parameterized neural networks and ∘ denotes element-wise multiplication. Then, the expectation over $a_{k}∼\pi$ can be converted to the expectation over the normal variable whose distribution is irrelevant to the states and net parameters. Additionally, the squashed Gaussian policy constrains the input voltages in $\left[0,V_{\max}\right]$. According to (36), the action given $s_{k}$, $c_{k}$ and $\zeta_{k}$ is written as $a_{k}=a_{\theta }\left(s_{k},c_{k},\zeta_{k}\right)$. Note that this is an invertible map between $a_{k}$ and $\zeta_{k}$. Therefore, we can compute the log-probabilities in closed form according to the change of the variable formula [55] as

(37) $$\begin{array}{cc} \log\left(\pi_{\theta }\left(a_{k}|s_{k},c_{k}\right)\right) & =\log\left(p(\zeta_{k})\right)-\log\left|\det J_{a_{\theta }}\left(\zeta_{k}\right)\right|\\ & =\log\left(p(a_{\theta }^{-1}\left(s_{k},c_{k},a_{k}\right))\right)+\log\left|\det J_{a_{\theta }^{-1}}\left(a_{k}\right)\right| \end{array}$$

where $a_{\theta }^{-1}$ is the inverse function of $a_{\theta }$ given $s_{k}$ and $c_{k}$, and *J* denotes the Jacobian matrix.

With $Q^{\pi_{\theta }}$ approximated by the minimum of the two critic networks, the loss function for the actor network is obtained as

(38) $$L\left(\theta \right)=\sum_{b\in B}\left(\min_{i=1,2}Q_{\phi_{i}}\left(s_{k},a_{\theta }\left(s_{k},c_{k},\zeta_{k}\right),c_{k},k\right)-\beta \log(\pi_{\theta }\left(a_{\theta }\left(s_{k},c_{k},\zeta_{k}\right)|s_{k},c_{k}\right)\right)$$

**Figure 4:**
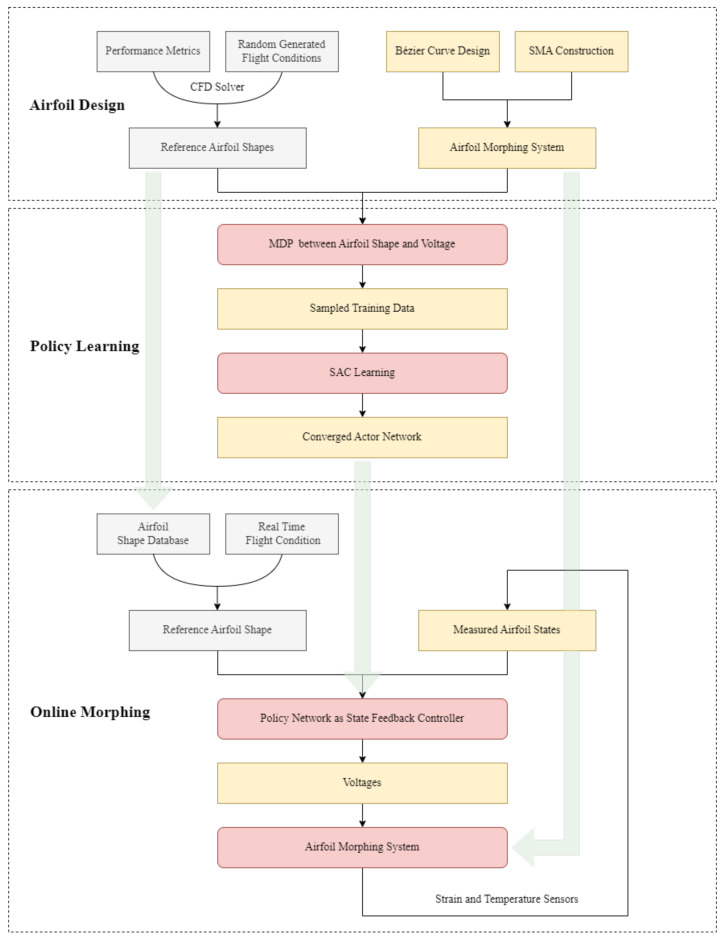
Overall flowchart of the proposed DRL-based airfoil morphing framework.

Then, we conduct the training by updating the actor and critic networks iteratively. The agent interacts with various randomly generated time-varying reference shape sequences to acquire training data, which faciliates the exploration and enables the policy to handle different morphing scenarios. When the training converges, we can use the actor network to calculate the required voltages and morph the airfoil to the reference shapes. Finally, the overall flowchart of the proposed morphing mechanism is given in Figure 4.

## 3. Results

In this section, a simulation is conducted to validate the proposed morphing method. The simulation is arranged in two stages. Firstly, we implement our method with random generated reference shapes and perform ablation studies to examine the superiorities brought by different parts of our algorithm. Subsequently, we apply the proposed method to track optimized airfoil shapes in different flight conditions and show the morphing procedures. The values of parameters in our simulation are given in Table 1 [40,41].

### 3.1. Tracking Random Shapes

In this stage, the superiorities of our method are illustrated in a variety of perspectives, including the state/action selection, reward configuration and entropy regularization. Without loss of generality, piece-wise constant trajectories of the radius factor for one control point are generated to represent the reference shapes. Then, our method is compared with three different settings of RL algorithms. The proposed method is denoted as RLM-SAC.

- Second-order state/action versus first-order state/action
  In Section 2, second-order MDP is adopted to model the hysteresis characteristics of the morphing system. Therefore, we chose the states and actions as combinations of that in current step and previous step. We compared the performance with RL algorithms where the policy is generated according to only current states, and the value function was also evaluated with only current states and actions as inputs that are applied in existing investigations on controling SMA wires. We refer to this as RLM-FO.
- Sparse reward versus squared error reward
  We designed a sparse reward taking value in $\left\{0,1\right\}$, which is different from traditional RL-based morphing research. We compared that with the square error rewards, which is given by
  (39) $$r_{k}^{\prime }=R\left(s_{k},c_{k}\right)=-\sum_{i=1}^{N}{\left|\gamma_{i,k}-\gamma_{i,k}^{\mathrm{ref}}\left(c_{k}\right)\right|}^{2}$$
  which is named RLM-SER.
- SAC versus DQN
  The entropy regularization improves the capability of exploration in our algorithm. A modified deep Q learning method was implemented as a comparison, where only the entropy loss was removed, and both the double-Q setting and reparameterization trick remained. We denote this as RLM-DQN.

All RL realizations were trained through 150 epochs, in each of which 5 episodes with 40 s of time and 200 time steps were executed. The critic network was constructed using multilayer perceptrons (MLP) of 3 hidden layers and 128 units per layer. The actor network adopted similar structures, where additional fully connected layers were attached to produce the mean and standard variations of the policy. The training was started with actions uniformly sampled from the valid action space bounded by $\left[0,V_{\max}\right]$ for 5000 steps to explore the state space sufficiently. Then, the networks were updated every step with a batch size of 200. A fixed learning rate was set as 0.002, and other hyperparameters used in RL training are shown in Table 2. The actions were generated from the stochastic policy in training phase but produced in a deterministic way according to the mean value of the actor network in the testing phase.

**Figure 5:**
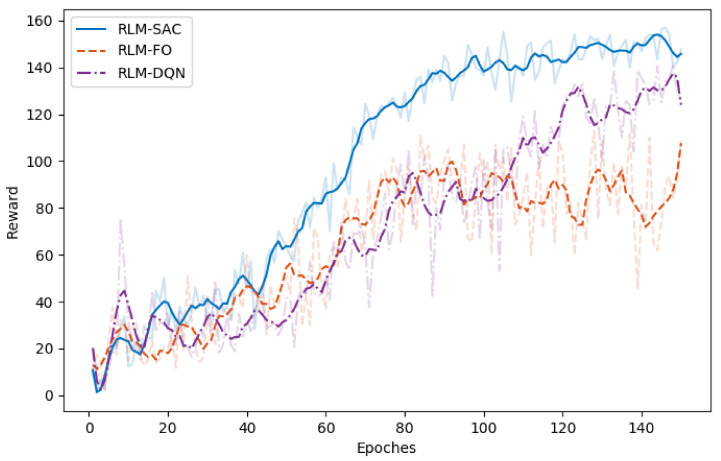
Reward of different RL realizations over the training trajectories. The data are smoothed via Savitzky–Golay filter with window size 15 and order 5.

**Figure 6:**
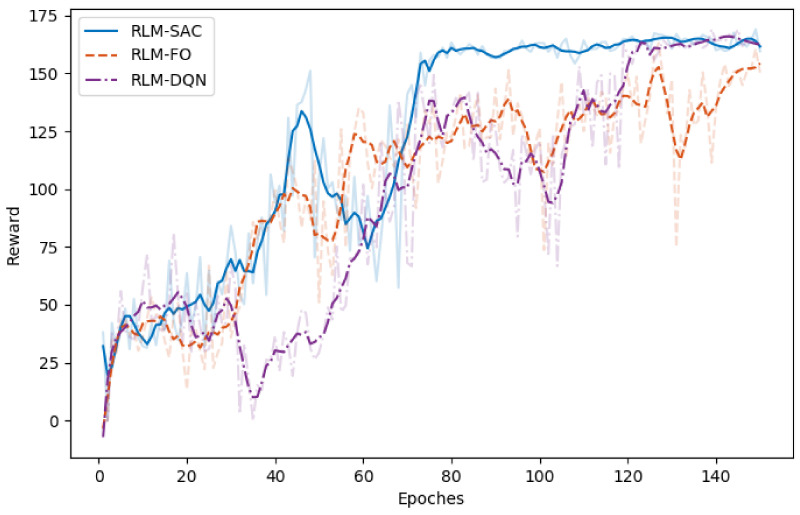
Reward of different RL realizations over the test trajectories. The data are smoothed via Savitzky–Golay filter with window size 15 and order 5.

**Figure 7:**
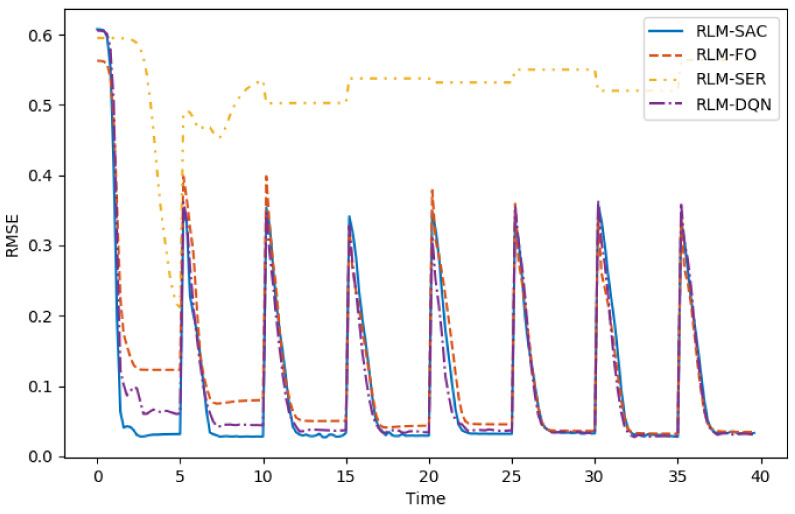
RMSE of radius factors generated by different RL realizations. The flight conditions in test trajectories change every 5 s, and at this time, the airfoil is expected to morph to a new reference shape.

**Figure 8:**
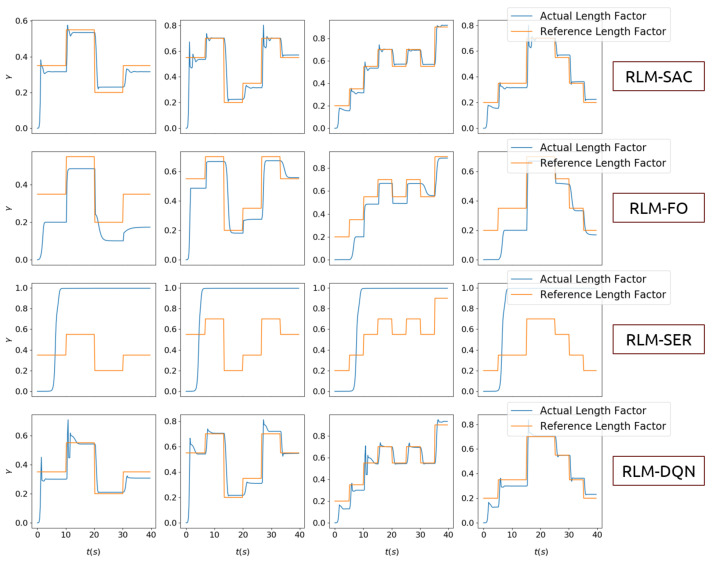
Illustration of some reference trajectories and the performance of each RL method.

The results are shown in Figure 5, Figure 6, Figure 7 and Figure 8. In Figure 5 and Figure 6, we illustrate the rewards acquired in the training and test trajectories, respectively, during the training procedure. A Savitzky–Golay filter [56] was adopted to smooth the data such that the values and trends of the rewards are illutstrated more clearly. Note that the reward of RLM-SER was not included because of a different reward setting. After the training was finished, the algorithms were executed on 100 random generated test reference trajectories. We present the root-mean-squared error (RMSE) of the radius factor through the flight time in Figure 7. Some of the trajectories and the corresponding results produced by the four RL realizations are depicted in Figure 8 for an intuitive comparison.

From the results, we can see the benefits of each important component in our algorithm. Firstly, RL using square error reward totally fails to produce effective actions in our environment, which is shown from both the RMSE and example trajectories. RLM-FO acquires inferior performance compared with RLM-SAC, especially in the temperature-switching procedure, which can be validated by the middle sections of the example trajectories. This is a result of the fact that the hysteresis cannot be characterized well by first-order states and actions. Lastly, RLM-DQN obtains better performance than RLM-FO and RLM-SRE, and it achieves a similar reward to RLM-SAC at the end of training. However, from the illustrations of rewards in Figure 5 and Figure 6, it is shown that RLM-DQN converges much slower than RLM-SAC. This is due to the improvement of exploration capability provided by entropy regularization.

### 3.2. Morphing Procedure Simulation

**Figure 9:**
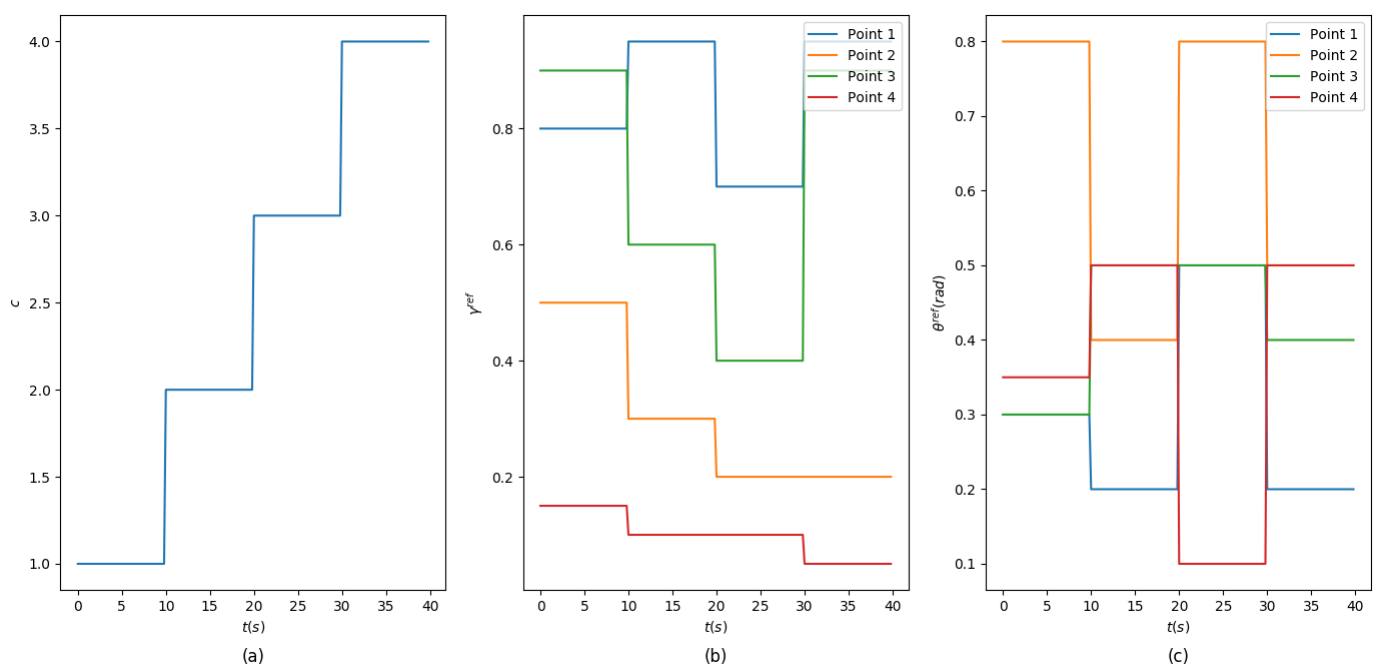
Trajectory of flight conditions. (**a**) Condition indexes. (**b**) Trajectories of optimal radius factor for each control point. (**c**) Trajectories of optimal angle for each control point.

In this stage, the trained actor network is applied to morph an airfoil controlled by four points in a given flight procedure with varying flight conditions. Since the focus of this work is morphing control, in each condition, the optimal shape is assumed to be solved in anticipation by shape optimization techniques and determined by the radii and angles of control points [14,18,20]. The average coefficient and scale coefficient are fixed as α=[0.12,0.4,0.4,0.12] and η=[0.5,0.5,0.5,0.5]. The trajectory of flight conditions and the corresponding parameters of optimal shapes are shown in Figure 9.

**Figure 10:**
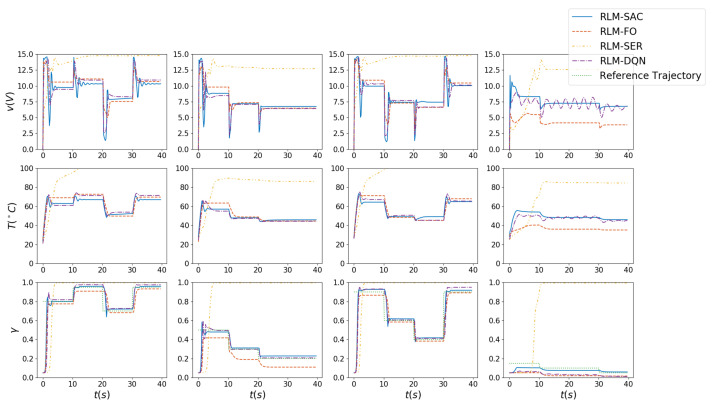
Voltages, temperatures and radius factors of control points. Each column represents the values of a point.

Since the position control technique of motors is relatively sufficiently developed, we assumed that the angles of the points are controlled by DC motors with accurately known linear dynamic models, which can drive the points to desired angles rapidly with subtle errors. Then we generate input voltages to heat the SMA wires and adjust the radius factors. The voltages, temperatures and radius factors of all points are summarized in Figure 10. It is shown that with constrained voltages, the wires can track the reference lengths well.

**Figure 11:**
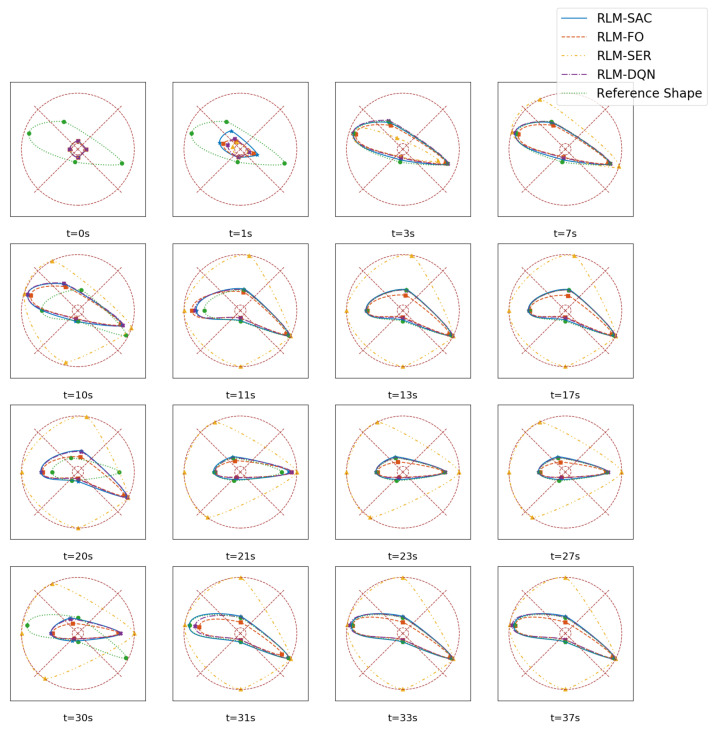
Illustration of the morphing procedure. Each row represents a morphing stage.

Furthermore, we illustrate the morphing procedures in Figure 11. We can see intuitively that with the proposed RLM-SAC method, the airfoil is capable of morphing into the optimized shape within about 3 s after encountering a new flight environment, which validates both the morphing accuracy and morphing speed.

Quantitative comparisons on the length factor differences and shape differences are given in Table 3. The average length factor differences are calculated according to the reference length factor and actual length factor as

(40) $$e_{\mathrm{length}}=\frac{1}{KN}\sum_{k=0}^{K}\sum_{i=1}^{N}\frac{\left|\gamma_{i,k}-\gamma_{i,k}^{\mathrm{ref}}\right|}{\gamma_{i,k}^{\mathrm{ref}}}$$

The difference between the actual and reference shapes are evaluated using distances between the control points. An average shape difference over all time is calculated as

(41) $$e_{\mathrm{shape}-\mathrm{avg}}=\frac{1}{KN}\sum_{k=0}^{K}\sum_{i=1}^{N}\left|\right|p_{i,k}-p_{i,k}^{\mathrm{ref}}{\left|\right|}_{2}$$

where $\left|\right|p_{i}-p_{i}^{\mathrm{ref}}{\left|\right|}_{2}$ is the $L_{2}$ distance between the two Cartesian coordinates $p_{i,k}$ and $p_{i,k}^{\mathrm{ref}}$. Additionally, the steady shape error is evaluated by

(42) $$e_{\mathrm{shape}-\mathrm{end}}=\frac{1}{4N}\sum_{t=1}^{4}\sum_{i=1}^{N}\left|\right|p_{i,k_{t}}-p_{i,k_{t}}^{\mathrm{ref}}{\left|\right|}_{2}$$

where $k_{t}$ denotes the end time step of each flight condition. It is shown that our method acquires the best performance on all metrics. The proposed RLM-SAC method provides an average 29.8% performance improvement over the second-best RLM-DQN method. The length differences and average shape differences, which are averaged over all time steps, demonstrate that our method can morph the airfoil into desired shapes more rapidly, while the steady shape difference validates the morphing accuracy. Lastly, in this work, we give the reference airfoil shape directly and focus on the morphing performance of the proposed method. It will be interesting and meaningful to combine the shape optimization task with the morphing control problem in the future.

## 4. Conclusions

In this work, a novel deep reinforcement learning-based morphing control method is proposed for an asymmetric morphing airfoil. The airfoil is designed via Bézier curves and is capable of morphing from a baseline shape to an asymmetric shape. The morphing mechanism is modeled via SMA wires, which adjust shape parameters, especially the radii of the control points. To actuate the SMA wires, resistive heating is performed, but the hysteresis characteristics between the SMA strain and temperature make the dynamic system nonlinear and non-Markovian, which brings difficulties to the design of the control algorithm and the RL framework. Therefore, hyperbolic tangent curves are adopted to model the strain-temperature relationship and derive a second-order MDP describing the system, which is then transformed into a valid MDP and provides guidance for the selection of states and actions. Based on the constructed MDP, we modify the SAC algorithm and develop an RL scheme where input voltages are generated to morph the airfoil instantaneously according to reference-optimized shapes. Lastly, ablation studies on random generated reference trajectories are conducted to demonstrate the benefits brought by different components of our RL implementations, and we perform simulations of morphing procedures to validate that our method is able to morph the airfoil into the optimized shapes rapidly and accurately. Future works include incorporating the aerodynamic performance optimization directly into the morphing control and exploiting learning-based morphing policies for more complicated bio-inspired morphing aircraft.

## Figures and Tables

**Table 1:** Values of system parameters used in simulations.

Parameter	Value	Parameter	Value
$m_{w}$	$1.14\times 10^{-4}$	$A_{w}$	$4.72\times 10^{-4}$
$c_{w}$	837.4	$R_{w}$	50.8
$T_{f}$	20	$h_{w}$	120
*H*	0.995	$c_{b}$	0.147
$c_{w}$	$1.25\times 10^{-5}$	$c_{s}$	0.001
$c_{tl}$	46	$c_{tr}$	65

**Table 2:** Values of hyperparameters used in RL.

Parameter	Value	Parameter	Value
ξ	0.98	α	0.2
ρ	0.995	$V_{\max}$	15
$r_{p}$	1	$r_{v}$	0.02
$e_{\mathrm{thr}}$	0.1		

**Table 3:** Quantitative comparisons of different morphing methods.

	RLM-SAC	RLM-FO	RLM-SER	RLM-DQN
$e_{\mathrm{length}}$	14.04%	31.44%	323.6%	23.24%
$e_{\mathrm{shape}-\mathrm{avg}}$	0.0533	0.0645	0.2202	0.0601
$e_{\mathrm{shape}-\mathrm{end}}$	0.0019	0.0055	0.0494	0.0031

## Data Availability

Not applicable.

## Abbreviations

The following abbreviations are used in this manuscript:

CFD	Computational Fluid Dynamics
MDP	Markov Decision Process
NACA	National Advisory Committee for Aeronautics
RL	Reinforcement Learning
RMSE	Root-Mean-Squared Error
SAC	Soft Actor-Critic
SMA	Shape Memory Alloy
UAV	Unmanned Aerial Vehicle
